# Evaluation and revision of core postoperative nursing outcomes for laryngeal carcinoma in China

**DOI:** 10.1186/s12912-021-00569-4

**Published:** 2021-03-23

**Authors:** Yong-Xia Ding, Hui Yang, Ya-Xuan Sun, Jinxia Xu, Li Jing, Yan Ning, Bin-Quan Wang

**Affiliations:** 1 Nursing College of Shanxi Medical University, Taiyuan City, Shanxi China; 2 Shanxi Key Laboratory of Otolaryngology, Head and Neck Cancer, Taiyuan City, Shanxi China; 3 Nursing Department of the First Hospital, Shanxi Medical University, Taiyuan City, Shanxi China; 4 Department of Neurology, Shanxi People’s Hospital, Taiyuan City, Shanxi China; 5 Shanxi Cancer Hospital, Taiyuan City, Shanxi China; 6 Department of Otolaryngology, Head and Neck Surgery, The First Hospital of Shanxi Medical University, Taiyuan City, Shanxi China

## Abstract

**Background:**

The core nursing outcomes for laryngeal carcinoma in China needed further screening and revision. This study aimed to evaluate and revise a questionnaire according to the “Core Nursing Outcomes for Otorhinolaryngology Head-Neck” of the Nursing Outcomes Classification (NOC, 5th Edition), and determine suitable postoperative nursing outcomes for patients with laryngeal carcinoma in China.

**Methods:**

The commonly used postoperative nursing outcomes for laryngeal carcinoma were screened using a questionnaire given to 93 nurses. An initial expert consultation questionnaire was constructed to discuss the indicators for each nursing outcome. A total of 20 experts were identified using the Delphi method, and their recommendations and revisions on the selected nursing outcomes were collected.

**Results:**

A total of 14 postoperative core nursing outcomes and 69 indicators were identified for postoperative patients with laryngeal carcinoma, which are subordinate to 4 domains of the NOC: “Physiologic Health”, “Psychosocial Health”, “Health Knowledge & Behavior”, and “Perceived Health”.

**Conclusions:**

The screening and revision of the NOC outcomes and indicators of the Delphi method could be applied to assess the effect of nursing intervention and the quality of the nursing service in China.

**Supplementary Information:**

The online version contains supplementary material available at 10.1186/s12912-021-00569-4.

## Background

Head and neck squamous cell carcinoma is one of the most common cancers, and includes tumors in the oral cavity, pharynx, hypopharynx, and larynx [1]. Laryngeal carcinoma constitutes approximately 1–5% of all malignant tumors and 11–22% of all head and neck cancer cases in China [2, 3]. In terms of treatment, chemoradiotherapy is often used for patients with early-stage laryngeal carcinoma, while surgery is the most common treatment strategy for laryngeal carcinoma at an advanced stage, including total and partial laryngectomies [4, 5]. However, the quality of life of patients that have had surgery is greatly impacted owing to the use of tubes and the loss of speech [6, 7]. Nowadays, numerous nursing strategies are being applied to clinical practice in order to improve respiratory functions, as well as pronunciation, swallowing, and social or psychological disorders caused by dyspnea and ostomy [8–10]. However, there was no comprehensive assessment tool to evaluate the effect of nursing care in China, and this should be developed to assess postoperative health outcomes for patients with laryngeal carcinoma.

The Nursing Outcomes Classification (NOC) is a comprehensive and standardized classification of patient and client outcomes, which makes it possible to identify and measure the outcomes of nursing care. Moreover, the patients’ follow-up progress is also assessed using the NOC. The NOC is designed and provided by nurses, which consists of 490 core nursing outcomes in 5th Edition [11] and 540 core nursing outcomes in 6th Edition [12], that measure a patient’s response to interventions. Each nursing outcome has a label name and assessment scale, and contains a list of indicators to assess the evaluated outcomes. This current study applied the NOC’s “Core Nursing Outcomes for Otorhinolaryngology Head-Neck” in 5th Edition for initial screening [11], and then revised both the core nursing outcomes and the indicators for patients with laryngeal carcinoma in China through a questionnaire investigation and expert consultation.

## Methods

### Study design

The most commonly used core postoperative nursing outcomes for patients with laryngeal carcinoma were identified from the 75 core nursing outcomes included in the “Core Nursing Outcomes for Otorhinolaryngology Head-Neck” using a questionnaire. The self-defined questionnaire was applied as an assessment tool, and information regarding the degrees of use of core nursing outcomes were collected. The degree of use of nursing outcomes were assessed on a 0–100% time scale that represented the percentage of time used for this outcome in care. A nursing outcome was selected as one of the core nursing outcomes if more than 50% of the time was spent, and it was reported by nurses at all 10 centers [13]. Following this, the indicators of each nursing outcome were screened, and then revised alongside their related indicators, through expert consultation using the Delphi expert consultation method [13].

### Screening in core nursing outcomes

The screening process refers to the approach of identifying the core nursing outcomes of the NOC, which is based on their degree of use. A total of 130 nurses in otorhinolaryngology from 10 hospitals (grade IIIA) in Shanxi, Beijing, Tianjin, Liaoning, and the Inner Mongolia Autonomous Region were selected using a convenience sampling approach. The inclusion criteria was a nurse practitioner that has at least 2 years of clinical nursing experience in otolaryngology head and neck surgery. Considering the cultural divergence and differences in habits between China and US, the current study refers to the criteria of Liu et al., which divided the frequency of use into 5 grades: never use, rarely use (< 1 time/week), less use (1 time/week), more use (several times/week), and often use (several times/week) [14].

The field investigation took place at hospitals in Shanxi province; first, the research term contacted the department of otolaryngology, and then the field investigation was conducted with nurses. The questionnaire was filled out and collected after the researcher introduced the purpose of the survey and explained the method for filling it out. The investigation approach in Beijing, Tianjin, Liaoning, and the Inner Mongolia Autonomous Region was to make contact through the department of otolaryngology via telephone, E-mail, or other forms of communication; following this, the questionnaires were sent to a controller and details of the purpose of the survey and the method for filling it out were clarified. After this, the controller organized all the selected nurses for field investigation, and all of the questionnaires were recycled by mail or scanned and sent electronically.

### Screening in indicators of core nursing outcomes

The NOC indicated that the core nursing outcomes consist of a variable number of care indicators, and the score of each outcome was defined as the sum of the scores of the indicators. The researcher assessed the outcome of patients based on various selected indicators or one single indicator, depending on the actual situation. The most commonly used core nursing outcomes for postoperative care of laryngeal carcinoma were already identified in the first step. We adopted the expert group method to screen the specific indicators based on the need to use them in the actual evaluation, due to the fact that there was duplication across the indicators. The group was set up and involved six experts, which consisted of 1 director and doctoral supervisor of otolaryngology head and neck surgery, 2 master’s supervisors, 1 deputy director and master’s supervisor in the nursing department, and 2 head nurses. The age of the experts ranged from 36 to 58 years old, their educational background was higher than the graduate level, and their title was higher than an associate senior. The team members evaluated 314 evaluation indicators “back to back”, and only two opinions were given for each indicator. After this, the results were summarized and discussed. The indicator was selected if more than 4 members agreed, and was deleted if less than 2 members agreed. If the indicator was agreed by 2–4 members, and the group discussion was performed by 6 experts. The indicator was selected when ≥4 of experts was agreed.

### Revision of core nursing outcomes and related indicators

The initially screened core nursing outcomes and related indicators were revised using the Delphi expert consultation method owing to the cultural divergence and differences in habits between China and US. The improved Delphi method of expert consultation was then applied to this study, and the number of consultations was terminated once an expert opinion was reached [13]. The results of expert consultation were analyzed using the expert positive coefficient, the degree of expert authority, the coordination degree of experts’ opinion, and the expert opinion concentration degree. The detailed process is listed as follows: (1) Selection of the experts: for the purpose of this study, the selection criteria for experts included having been engaged in clinical nursing or medical practice in the field of otolaryngology, with more than 10 years of work experience, a level of education greater than a bachelor’s degree, intermediate or more senior professional titles, volunteering to participate in the consultation, and filling out and providing feedback on the questionnaire in a timely manner. The number of experts that were consulted in the Delphi method always ranged from 12 to 30 [15]. In this study, a total of 20 nurse specialists from grade A hospitals were selected; (2) Expert consultation questionnaire: The Delphi expert consultation questionnaire was created based on the consultation table of postoperative core nursing outcomes for patients with laryngeal carcinoma. Each core nursing outcome and its related indicator was graded using a 5 point Likert scale (5: very important; 4: important; 3: general; 2: unimportant; and 1: very unimportant). The experts could list any modification, supplementary information, or other comments in the column of revised opinion, including additional items or other opinions. Moreover, the general characteristics of experts, their familiarity, and any potential impact factors were also summarized in the Expert self-assessment table; (3) Expert consultation process: Two rounds of expert consultations were conducted for the purpose of this study via E-mail. The duration of each round of consultation was 2 weeks, and reminders were sent to the experts after 1 week. The degree of completion of the questionnaire was checked after receiving an expert’s reply. Each expert was requested to complete any part of the questionnaire that was left unanswered. Statistical analysis and sorting was conducted after each round of questionnaire collection, and, then, the summarized results were fed back to the experts once the next round of the questionnaire had been issued; the consultation was completed if an agreement of opinion was reached among the experts.

### Data collation and statistical analysis

All collected data were independently entered into Excel by 2 researchers, and any conflicts between the researchers was settled by an additional researcher who referred to the original questionnaire. The general characteristics of sex, age, years of work experience, educational background, position, and professional title were presented as frequency, proportion, mean, and standard deviation. The core nursing outcomes and related indicators were assessed using the mean, standard deviation, and coefficient of variation. All of analyses in this study were conducted using version 17.0 of the IBM SPSS Statistics for Windows.

## Results

### Screening results for core nursing outcomes and related indicators

A total of 130 questionnaires were sent out and 93 were recovered, resulting in a 71.5% recovery rate. The use degree of 75 core nursing outcomes in “Core Nursing Outcomes for Otorhinolaryngology Head-Neck” was assessed using the following frequencies: never use, rarely use (< 1 time/week), less use (1 time/week), more use (several times/week), and often use (several times/week). By referring to the relationship between the sample size and the number of variables in the scale formulation, the first 18 core nursing outcomes with high scores (93/5 = 18.6) were obtained as the initial results (Table 1). A total of 129 indicators were identified among the 314 indicators related to the 18 core nursing outcomes, which then constituted the content of the Delphi expert consultation questionnaire.

**Table 1:** The 18 postoperative core nursing outcomes screened initially for patients with laryngeal carcinoma

Outcome	Coding	Total score	Mean	Standard deviation
Communication	0902	431	4.634	0.700
Nutritional Status	1004	393	4.226	1.369
Swallowing Condition: Pharyngeal Phase	1013	388	4.172	1.300
Respiratory Status: Airway Patency	0410	385	4.140	1.357
Respiratory Status: Ventilation	0403	385	4.140	1.164
Infection Severity	0703	383	4.118	1.359
Vital Signs	0802	383	4.118	1.451
Respiratory Status	0415	382	4.108	1.433
Nutritional status: Biochemical Measures	1005	368	3.957	1.652
Tissue Integrity: Skin & Mucous Membranes	1101	366	3.935	1.443
Will to Live	1206	358	3.849	1.406
Acceptance: Health Status	1300	352	3.785	1.473
Smoking Cessation Behavior	1625	346	3.720	1.512
Knowledge: Infection Management	1842	346	3.720	1.498
Pain Level	2102	346	3.720	1.473
Aspiration Preventing	1918	345	3.710	1.583
Risk Control: Tobacco Use	1906	344	3.699	1.301
Adaptation to Physical Disabilities	1308	336	3.613	1.460

### Representativeness and reliability of consulted experts

The general characteristics of the selected experts are presented in Table 2. The effective questionnaire recovery rate could reflect the degree of experts’ enthusiasm and their concern for this topic. All 20 experts responded to two rounds of expert consultation, and the effective questionnaire recovery rate was 100%, which suggested that the expert positive coefficient was high in this study. The degree of expert authority was measured by judgment (Ca) and expert familiarity (Cs), Cr = (Ca + Cs)/2. The degree of expert authority was between 0.70–0.95, and the overall expert authority degree was 0.805, which suggested that the experts were highly authoritative in this study (Table 3). The Kendall’s W value was applied to assess the coordination degree of expert opinion, and the W value ranged from 0 to 1. The W value was 1 and thus indicated the highest possible degree of coordination of expert opinion. The Kendall’s W was 0.11 and 0.77 in the two rounds of expert consultation, and significant results were detected, which indicated that the results were credible (Table 4). The degree of expert opinion concentration was presented as the mean, standard deviation, and coefficient of variation. The high degree of expert opinion concentration was considered large mean, small standard deviation, and coefficient of variation. The mean value of the coefficient of variation for each item gradually decreased after two rounds of consultation. The coefficient of variation of the overall importance in the first round of expert consultation was 0.14, while it was 0.11 in the second round of expert consultation (Table 5).

**Table 2:** The general characteristics of the identified experts

Variable	Group	Frequency	Proportion (%)
Sex	Male	5	25.0
	Female	15	75.0
Age (years)	30–39	8	40.0
	40–49	10	50.0
	≥ 50	2	10.0
Years of working	10–20	13	65.0
	21–30	6	30.0
	≥ 31	1	5.0
Professional title	Senior	8	40.0
	Association senior	12	60.0
Educational background	Undergraduate	10	50.0
	Master	7	35.0
	Doctor	3	15.0
Professional field	Clinical nurse	12	60.0
	Nursing supervision	4	20.0
	Nursing education	2	10.0
	Nursing research	2	10.0

**Table 3:** The details regarding expert authority degree

Expert	Cs	Ca					Expert authority degree
		Work experience	Theoretical analysis	Reference to literature	Intuition	Total scores for Ca	
1	1	0.50	0.20	0.10	0.10	0.90	0.95
2	0.80	0.50	0.20	0.10	0.10	0.90	0.85
3	0.80	0.50	0.20	0.10	0.10	0.90	0.85
4	0.80	0.50	0.20	0.10	0.10	0.90	0.85
5	0.80	0.50	0.10	0.10	0.10	0.80	0.85
6	0.80	0.50	0.10	0.10	0.10	0.80	0.80
7	0.80	0.40	0.30	0.10	0.10	0.90	0.85
8	0.80	0.40	0.30	0.10	0.10	0.90	0.85
9	0.80	0.50	0.10	0.10	0.10	0.80	0.80
10	0.80	0.50	0.10	0.10	0.10	0.80	0.80
11	0.80	0.50	0.10	0.10	0.10	0.80	0.80
12	0.80	0.50	0.10	0.10	0.10	0.80	0.80
13	0.80	0.40	0.20	0.10	0.10	0.80	0.80
14	0.80	0.40	0.20	0.10	0.10	0.80	0.80
15	0.80	0.40	0.10	0.10	0.10	0.70	0.75
16	0.80	0.40	0.10	0.10	0.10	0.70	0.75
17	0.80	0.40	0.10	0.100	0.10	0.70	0.75
18	0.80	0.40	0.10	0.10	0.10	0.70	0.75
19	0.80	0.40	0.10	0.10	0.10	0.70	0.75
20	0.50	0.40	0.30	0.10	0.10	0.90	0.70
	0.795					0.81	0.805

**Table 4:** Kendall’s W value of two rounds of expert consultations

Rounds	Number of items	W	χ^2^	*P*
Round 1	147	0.11	141.57	< 0.001
Round 2	83	0.77	801.78	< 0.001

**Table 5:** The egree of expert opinion concentration regarding core nursing outcomes

NO.	nursing outcome	Round 1			Round 2		
		Mean	SD	CV (%)	Mean	SD	CV (%)
A	0802 Vital Signs	4.85	0.36	7.36	4.95	0.22	4.40
B	0415 Respiratory Status	4.90	0.30	6.12	4.90	0.30	6.12
C	0403 Respiratory Status: Ventilation	4.80	0.40	8.33	/	/	/
D	0401 Respiratory Status: Airway Patency	4.70	0.46	9.75	/	/	/
E	0703 Infection Severity	4.70	0.46	9.75	4.75	0.43	9.12
F	0902 Communication	4.90	0.30	6.12	4.90	0.30	6.12
G	1013 Swallowing Condition: Pharyngeal Phase	4.90	0.30	6.12	4.90	0.30	6.12
H	1004 Nutritional Status	4.90	0.30	6.12	4.90	0.30	6.12
I	1005 Nutritional status: Biochemical Measures	4.45	0.74	16.63	/	/	/
J	1101 Tissue Integrity: Skin & Mucous Membrane	4.50	0.74	16.48	4.50	0.74	16.48
K	1300 Acceptance: Health Status	4.60	0.73	15.98	4.60	0.73	15.97
L	1206 Will to Live	4.55	0.74	16.26	4.70	0.46	9.75
M	1308 Adaptation to Physical Disabilities	4.60	0.73	15.98	4.65	0.65	14.06
N	1842 Knowledge: Infection Management	4.75	0.43	9.12	4.75	0.43	9.12
O	1625 Smoking Cessation Behavior	4.90	0.30	6.12	4.90	0.30	6.12
P	1906 Risk Control: Tobacoo Use	4.90	0.30	6.12	/	/	/
Q	1918 Aspiration Preventing	4.95	0.21	4.40	4.95	0.22	4.40
R	2102 Pain Level	4.05	0.67	16.52	4.35	0.48	10.96

### Results of core nursing outcomes

The screening criteria for the core nursing outcomes in this study was the mean value of items being more than 4.0. The overall index score less than - S was considered as a deleted criterion for level 2 items. In the first round of expert consultation, all 18 core nursing outcomes were retained in accordance with the inclusion criteria (Table 1). Eight experts proposed that “Respiratory Status” should be retained in the outcomes of Respiratory Status”, “Respiratory Status: Airway Patency”, and “Respiratory Status: Ventilation” because of the repeatability of the evaluation indicators. Six experts suggested removing either “Smoking Cessation Behavior” or “Risk Control: Smoking”. Two experts mentioned that “Nutritional Status: Biochemical Measures” should be removed because of the large number of items in routine laboratory examinations after laryngeal carcinoma surgery. After this, the 18 core nursing outcomes were adjusted to 14 after excluding “Respiratory Status: Airway Patency”, “Respiratory Status: Ventilation”, “Risk Control: Smoking”, and “Nutritional Status: Biochemical Measures”. Two experts suggested changing the definition of “Smoking Cessation Behavior” from “Personal actions to eliminate tobacco use” into “Individual measures taken to quit smoking”. For “Knowledge: Infection Management”, the definition of the “Extent of understanding conveyed regarding infection” was changed into “Knowledge of infection prevention and control”. For the outcome of “Vital Signs”, experts suggested using 080201 body temperature, 080203 pulse, 080204 respiration, 080205 systolic blood pressure, and 080206 diastolic blood pressure. For the outcome of “Respiratory Status”, the indicators of “041503 Depth of inspiration”, “040329 Asymmetrical chest expansion”, “040333 Distorted voice sounds on auscultation”, and “040334 Atelectasis” were deleted owing to a score of less than 4. Meanwhile, the experts also suggested adding the indicator of “040330 Impaired vocalization” into the outcome of “Communication”. For the outcome of “Communication”, the indicator of “090205 Use non-verbal language” was removed owing to the score of less than 4. One expert combined “090201 Use of written language”, “090203 Use of pictures and drawings”, and “090204 Use of sign language” into one indicator. Five experts proposed adding the indicator of “101302 Changes in voice quality” in “Swallowing condition” into the “Communication”. According to experts’ suggestions, we adjusted the indicator of “090201 Use of written language” to “Use of written words, pictures, or symbols”, while also adding “Impaired vocalization” and “Changes in voice quality”. For the outcome of “Nutritional Status”, experts indicated the intake of nutrients should include food and liquid intake, resulting in the indicators of “100402 Food intake” and “100408 Liquid intake” being deleted (owing to a score of less than 4). The indicator of “110,103 Elasticity” in the outcome of “Tissue Integrity: Skin and Mucous Membranes” was excluded. The indicator of “130013 Reports sense of life being worth living” in the outcome of “Acceptance: Health Status” was removed, and the outcome of “Adapt to Physical Disabilities” was added into “Acceptance: State of Health”. However, we retained “Adapt to Physical Disabilities” as a core nursing outcome based on the definition of outcomes and indicators; the indicator of “130803 Adapts to functional limitations” was selected and entered in the second round of expert consultation. For the outcome of “Knowledge: Infection Management”, the indicators of “184206 Monitoring procedures for infection”, “184208 Action to increase resistance to infection”, “184210 Follow-up for diagnose infection”, “184211 Signs and symptoms for exacerbation of infection”, and “184221 Influences of nutrition on infection” were excluded. For the outcome of “Smoking Cessation Behavior”, the indications of “162501 Express willingness to stop smoking”, “162503 Identifies benefits of smoking cessation”, “162514 Obtains assistance from health professional”, “162516 Uses reputable sources of information”, “162521 Uses prescription medication as recommended”, “162522 Uses non-prescription medications as recommended”, “162518 Uses alternative therapies”, “162524 Uses available community resources”, “162525 Participates in counseling” should have been removed owing to scores of less than 4. Finally, the indicator of “191806 Selects foods or drinks of proper texture” in the outcome of “Aspiration Preventing” was excluded.

In the second round of expert consultation, the approval rate for the 14 identified core nursing outcomes was 100%, and the score for each outcome was greater than 4 (Table 6). All indicators in the 14 identified core nursing outcomes were greater than - S, and were thus retained (Supplemental 1). After two rounds of expert consultation, a total of 14 core nursing outcomes, containing a total of 69 indicators, were identified, which involved the following 4 fields: “Physiologic Health”, “Psychosocial Health”, “Health Knowledge & Behavior”, and “Perceived Health” (Table 6).

**Table 6:** The details regarding postoperative core nursing outcomes and related indicators for patients with laryngeal carcinoma

Field	Outcome	Indicator
Physiologic Health	0802 Vital Signs	080201 Body temperature
		080203 Pulse
		080204 Respiration
		080205 Systolic blood pressure
		080206 Diastolic blood pressure
	0415 Respiratory Status	041501 Respiratory rate
		041502 Respiratory rhythm
		041532 Airway patency
		041514 Dyspnea at rest
		040331 Accumulation of sputum
		040208 PaO2
		040209 PaCO2
		040309 Accessory muscle use
	0703 Infection Severity	070303 Full-smelling discharge
		070304 Purulent sputum
		070305 Purulent drainage
		070334 Tenderness
		070319 Chest X-ray infiltration
		070326 White blood count elevation
	0902 Communication	090201 Use written language
		090202 Use of spoken language
		040330 Impaired vocalization
		101,302 Changes in voice quality
	1013 Swallowing Condition: Pharyngeal Phase	101,301 Timely swallow reflex
		101,304 Number of swallows appropriate for bolus size
		101,303 Choking
		101,314 Coughing
		101,315 Gagging
		101,306 Increased swallow effort
		101,310 Nasal reflux
		101,316 Aspirations
	1004 Nutritional Status	100,401 Nutrient intake
		100,403 Energy
		100,405 Weight/height ratio
	1101 Tissue Integrity: Skin & Mucous Membrane	110,101 Skin temperature
		110,102 Sensation
		110,113 Skin integrity
		110,117 Scar tissue
		110,124 Induration
		110,125 Corneal abrasion
Psychosocial Health	1300 Acceptance: Health Status	13,008 Recognizes reality of health situation
		130,009 Pursues information about health
		130,010 Copes with health situation
		130,014 Performs self-care tasks
	1206 Will to Live	120,602 Expression of hope
		120,603 Expression of optimism
		120,613 Use of treatments to lengthen life
		120,609 Use of strategies to enhance health
	1308 Adaptation to Physical Disabilities	130,803 Adapts to functional limitations
Health Knowledge & Behavior	1842 Knowledge: Infection Management	184,204 Signs and symptoms of infection
		184,207 Importance of hand sanitation
		184,209 Treatment for diagnosed infection
		184,219 Risk of drug resistance
	1625 Smoking Cessation Behavior	162,502 Express belief in the ability to stop smoking
		162,504 Identifies negative consequences of tobacco use
		162,505 Develop effective strategies to eliminate tobacco use
		162,523 Use available support groups
	1918 Aspiration Preventing	191,801 Identify risk factors
		191,802 Avoid risk factors
		191,803 Positions self-upright for eating and drinking
		191,804 Selects foods according to swallowing ability
		191,805 Positions self on side for eating and drinking as need
		191,808 Uses liquid thickeners as needed
		191,809 Maintains oral hygiene
		191,810 Remains upright for 30 min after eating
Perceived Health	2102 Pain Level	21,024 Length of pain episodes
		210,221 Rubbing affected area
		210,206 Facial expressions of pain
		210,225 Tearing

## Discussion

The current study was based on the concept and framework of the 5th Edition of NOC, and applied the Delphi expert consultation method to evaluate and revise the core postoperative nursing outcomes and related indicators for Chinese patients with laryngeal carcinoma. The results found the expert positive coefficient, degree of expert authority, coordination degree of experts’ opinion, and expert opinion concentration degree to be high. Moreover, a total of 14 core nursing outcomes containing 69 indicators in the domains of “Physiologic Health”, “Psychosocial Health”, “Health Knowledge & Behavior”, and “Perceived Health” were identified after 2 rounds of the expert consultation method. Therefore, the revision of the core nursing outcomes and their related indicators should be recommended for patients with laryngeal carcinoma.

### Screening methods for core nursing outcomes

The initial screening method for core nursing outcomes in this study referred to the Center for Nursing Classification of the College of Nursing at the University of Iowa through the use of outcomes by nurses in the field of “Core Nursing Outcomes for Otorhinolaryngology Head-Neck”, which consists of 75 core nursing outcomes [11]. Although the evaluation and revision of the NOC in other fields has already been illustrated, there was no study revision of the NOC in otolaryngology head and neck surgery in China [16–19]. The current study used the otolaryngology clinical specialty nurses as respondents, as they are the main implementers of nursing measures and could directly evaluate the effect of nursing intervention. However, we found that some clinical front-line nurses were not familiar with the nursing outcomes, and there were still doubts about the names and definitions of outcomes, which required further explanation by researchers. This indicated that the NOC was not promoted or popularized in China, in contrast to the perfect NOC databases that have been established in many other countries. The potential reason for this could be related to the lack of managerial support, personnel training, and information technology of NOC in China [20]. The results of this study could promote the cognition and understanding of NOC among nursing staff in China.

### Revised content regarding core nursing outcomes and related indicators

The results of this study involved 14 core nursing outcomes, 8 in the domain of “Physiologic Health”, 2 in the domain of “Psychosocial Health”, 3 in the domain of “Health Knowledge & Behavior”, and the remaining 1 in the domain of “Perceived Health”, which could comprehensively highlight the nursing problems and intervention directions for patients after surgery. Firstly, the outcomes in “Physiologic Health” were most important for patients after surgery, including postoperative respiratory tract management, eating, swallowing, pronunciation, infection management, and pain control. Secondly, for the domain of “Psychosocial Health”, the patients’ pronunciation and appearance could be affected by laryngeal surgery, which could cause great difficulties for interpersonal communication and social adaption. Moreover, postoperative anxiety, depression, and social adaptation were also common, and could affect the prognosis for patients after laryngeal surgery [21]. The outcomes of “Acceptance: Health Status”, “Will to Live”, and “Adaptation to Physical Disabilities” could reflect the results of psychological nursing intervention. Thirdly, in the domain of “Health Knowledge & Behavior”, the acquisition of patients’ health knowledge and behavior could improve the promotion of health and health education, which could affect patients’ postoperative self-management ability, rehabilitation level, and quality of life. Postoperative respiratory tract infection, eating cough, and aspiration are the most common complications in patients after laryngeal surgery. Moreover, some serious complications, such as tube removal, were associated with an increased risk of mortality, which require patients or family caregivers to have adequate recognition and emergency treatment ability. Furthermore, smoking is significantly associated with the progression and prognosis of laryngeal carcinoma, and should be stopped to improve the nursing outcome after surgery [22]. In addition, the nursing of tracheal cannula, oral care, and knowledge of pronunciation was also important for patients, although no related core nursing outcome was eligible in this study. One potential reason for this could be the nurses’ inadequate understanding of the definition of “Knowledge: Treatment Procedure” during the initial screening process. Finally, for the domain of “Perceived Health”, postoperative pain is the most common symptom of patients after surgery, and should thus be considered the focus of nursing. These results suggest that nursing care for patients after laryngeal surgery should focus on the maintenance of physiological functions and health education, as well as enhancing the intervention and evaluation of patients’ psychological disorders.

### Comparison of the core nursing outcomes in China and the US

The 75 Core nursing outcomes in the NOC were 100% used in the “Core Nursing Outcomes for Otorhinolaryngology Head-Neck”, as identified by the Otolaryngology and Head and Neck Nurses Association. Considering the cultural divergence and differences in habits between China and US, each indicator in self-defined questionnaire was divided the frequency of use into 5 grades: never use, rarely use (< 1 time/week), less use (1 time/week), more use (several times/week), and often use (several times/week) [14]. Moreover, all of initially screened core nursing outcomes and related indicators were revised using the Delphi expert consultation method [13]. When surveying 93 otolaryngology nurses, this study found no core nursing outcome with score rate of 100%; the top five outcomes were “Communication”, “Nutritional Status”, “Swallowing Condition: Pharyngeal Phase”, “Respiratory Status: Airway Patency”, and “Respiratory Status: Ventilation”. The above nursing outcomes could reflect the physiological functions of the larynx being affected by surgery, including pronunciation, swallowing, and breathing. A preventive tracheotomy should be applied to prevent breathing difficulties caused by local swelling and blood flowing into the respiratory tract for patients after a laryngectomy. Airway management, nutritional support, and oral care should also be considered key nursing points for patients after surgery, to prevent respiratory tract infection, cough, use nasogastric, and inability to make sounds [21]. The outcomes of “Respiratory Status”, “Nutritional Status”, “Swallowing Condition”, and “Communication” were related to the above nursing outcomes, and should be regarded as the most common nursing outcomes. The outcome of “Vital Signs” was widely used in clinical practice, although it did not get full marks in our study. A potential reason for this could be that the specialty of “Vital Signs” was not strong, and received inadequate attention from clinical nurses. Of the top 18 core nursing outcomes, 10 outcomes related to “Physiologic Health”, 3 outcomes related to “Psychosocial Health”, 4 outcomes related to “Health Knowledge & Behavior”, and 1 outcome related to “Perceived Health”. The core nursing outcomes from the NOC’s “Core Nursing Outcomes for Otorhinolaryngology Head-Neck” involved six health fields, while the outcomes of community health and functional health fields were not included in this study. The reason for this could explained by: (1) the score of community health being lower than expected because nurses in our study did not contact with the nursing outcomes in the community health field; and (2) the indicators of “out of bed activity”, “gait”, “activity endurance”, and “activity level” in the functional health field were not commonly used for patients recovering from laryngeal surgery.

## Conclusions

The current study constructed an evaluation index system of core nursing outcomes for patients after laryngeal surgery, which was based on the concept and framework of the 5th Edition of the NOC, and used the Delphi expert consultation method. The results of this study included 14 core nursing outcomes and 69 related indicators in the domains of “Physiologic Health”, “Psychosocial Health”, “Health Knowledge & Behavior”, and “Perceived Health”. These core nursing outcomes and indicators should be applied to assess the effect of nursing intervention and the quality of the nursing service for Chinese patients with laryngeal carcinoma.

## Supplementary Information

**Additional file 1: Supplement 1**. The related indicators in core nursing outcomes for patients after surgery

## Data Availability

The datasets used and/or analysed during the current study are available from the corresponding author on reasonable request.

## Abbreviations

NOC: Nursing Outcomes Classification
