# Supplementary material for: Evaluation and revision of core postoperative nursing outcomes for laryngeal carcinoma in China
Source: BMC Nurs. 2021 Mar 23;20:51. doi: 10.1186/s12912-021-00569-4 (PMC7989408; doi:10.1186/s12912-021-00569-4)
Supplement: Supplementary file 1 — Additional file 1: Supplement 1. The related indicators in core nursing outcomes for patients after surgery [file 12912_2021_569_MOESM1_ESM.docx]

Supplement 1. The related indicators in core nursing outcomes for patients after surgery

| Indicator | Round 1 | | | | | | Round 2 | | | | | |
| --- | --- | --- | --- | --- | --- | --- | --- | --- | --- | --- | --- | --- |
|  | Mean | SD | CV (%) | Score (%) | Overall score: (Mean* score/CV) | Excluded | Mean | SD | CV (%) | Score (%) | Overall score: (Mean* score/CV) | Excluded |
| A1 | 4.95 | 0.22 | 4.40 | 95 | 106.88 |  | 4.95 | 0.22 | 4.40 | 95 | 106.88 |  |
| A2 | 4.95 | 0.22 | 4.40 | 95 | 106.88 |  | 4.95 | 0.22 | 4.40 | 95 | 106.88 |  |
| A3 | 4.95 | 0.22 | 4.40 | 95 | 106.88 |  | 4.95 | 0.22 | 4.40 | 95 | 106.88 |  |
| A4 | 4.95 | 0.22 | 4.40 | 95 | 106.88 |  | 4.95 | 0.22 | 4.40 | 95 | 106.88 |  |
| A5 | 4.90 | 0.30 | 6.12 | 90 | 72.06 |  | 4.90 | 0.30 | 6.12 | 90 | 72.06 |  |
| B1 | 4.75 | 0.43 | 9.12 | 75 | 39.06 |  | 4.75 | 0.43 | 9.11 | 75 | 39.11 |  |
| B2 | 4.70 | 0.46 | 9.75 | 70 | 33.74 |  | 4.75 | 0.43 | 9.11 | 70 | 36.50 |  |
| B3 | 4.65 | 0.57 | 12.31 | 70 | 26.44 |  | 4.65 | 0.57 | 12.31 | 70 | 26.44 |  |
| B4 | 4.65 | 0.48 | 10.26 | 65 | 29.46 |  | 4.65 | 0.48 | 10.26 | 65 | 29.46 |  |
| B5 | 4.65 | 0.48 | 10.26 | 65 | 29.46 |  | 4.65 | 0.48 | 10.26 | 65 | 29.46 |  |
| B6 | 4.60 | 0.58 | 12.68 | 65 | 23.58 |  | 4.65 | 0.48 | 10.26 | 65 | 29.46 |  |
| B7 | 4.45 | 0.50 | 11.18 | 55 | 21.89 |  | 4.45 | 0.50 | 11.18 | 45 | 17.91 |  |
| B8 | 4.30 | 0.56 | 12.94 | 35 | 11.63 |  | 4.35 | 0.48 | 10.96 | 35 | 13.89 |  |
| C1 | 3.75 | 0.54 | 14.30 | 5 | 1.31 | √ |  |  |  |  |  |  |
| D1 | 3.95 | 0.80 | 20.37 | 30 | 5.82 | √ |  |  |  |  |  |  |
| D2 | 3.50 | 0.87 | 24.74 | 10 | 1.41 | √ |  |  |  |  |  |  |
| D3 | 3.40 | 0.86 | 25.30 | 10 | 1.34 | √ |  |  |  |  |  |  |
| E1 | 3.45 | 0.80 | 23.32 | 5 | 0.74 | √ |  |  |  |  |  |  |
| E2 | 3.55 | 0.86 | 24.35 | 15 | 2.19 | √ |  |  |  |  |  |  |
| E3 | 4.80 | 0.40 | 8.33 | 80 | 46.10 |  | 4.80 | 0.40 | 8.33 | 80 | 46.10 |  |
| E4 | 4.65 | 0.48 | 10.26 | 65 | 29.46 |  | 4.65 | 0.48 | 10.26 | 65 | 29.46 |  |
| E5 | 4.70 | 0.46 | 9.75 | 70 | 33.74 |  | 4.70 | 0.46 | 9.75 | 70 | 33.74 |  |
| E6 | 4.60 | 0.58 | 12.68 | 65 | 23.58 |  | 4.60 | 0.58 | 12.68 | 65 | 23.58 |  |
| E7 | 4.55 | 0.50 | 10.93 | 55 | 22.90 |  | 4.55 | 0.50 | 10.93 | 55 | 22.90 |  |
| E8 | 4.60 | 0.50 | 10.65 | 60 | 25.92 |  | 4.60 | 0.49 | 10.65 | 60 | 25.92 |  |
| F1 | 4.55 | 0.60 | 12.96 | 60 | 21.06 |  | 4.60 | 0.49 | 10.65 | 60 | 25.92 |  |
| F2 | 4.65 | 0.48 | 10.26 | 65 | 29.46 |  | 4.65 | 0.48 | 10.26 | 65 | 29.46 |  |
| F3 | 4.60 | 0.49 | 10.65 | 60 | 25.92 |  | 4.60 | 0.49 | 10.65 | 60 | 25.92 |  |
| F4 | 4.50 | 0.50 | 11.11 | 50 | 20.25 |  | 4.50 | 0.50 | 11.11 | 50 | 20.25 |  |
| F5 | 3.35 | 0.85 | 25.46 | 10 | 1.32 | √ |  |  |  |  |  |  |
| G1 | 3.50 | 0.81 | 23.03 | 10 | 1.52 | √ |  |  |  |  |  |  |
| G2 | 4.70 | 0.46 | 9.75 | 70 | 33.74 |  | 4.70 | 0.46 | 9.75 | 70 | 33.74 |  |
| G3 | 4.60 | 0.58 | 12.68 | 65 | 23.58 |  | 4.65 | 0.48 | 10.25 | 65 | 29.49 |  |
| G4 | 4.55 | 0.50 | 10.93 | 55 | 22.90 |  | 4.55 | 0.50 | 10.93 | 55 | 22.90 |  |
| G5 | 4.60 | 0.49 | 10.65 | 60 | 25.92 |  | 4.60 | 0.49 | 10.65 | 60 | 25.92 |  |
| G6 | 4.70 | 0.46 | 9.75 | 70 | 33.74 |  | 4.70 | 0.46 | 9.75 | 70 | 33.74 |  |
| G7 | 4.60 | 0.58 | 12.68 | 65 | 23.58 |  | 4.60 | 0.58 | 12.68 | 65 | 23.58 |  |
| G8 | 4.55 | 0.50 | 10.93 | 55 | 22.90 |  | 4.55 | 0.50 | 10.93 | 55 | 22.90 |  |
| G9 | 4.60 | 0.49 | 10.65 | 60 | 25.92 |  | 4.60 | 0.50 | 10.65 | 60 | 25.92 |  |
| H1 | 4.70 | 0.46 | 9.75 | 70 | 33.74 |  | 4.70 | 0.46 | 9.75 | 70 | 33.74 |  |
| H2 | 3.70 | 0.78 | 21.00 | 20 | 3.52 | √ |  |  |  |  |  |  |
| H3 | 3.75 | 0.77 | 20.44 | 20 | 3.67 | √ |  |  |  |  |  |  |
| H4 | 4.60 | 0.49 | 10.65 | 60 | 25.92 |  | 4.60 | 0.49 | 10.64 | 60 | 25.94 |  |
| H5 | 4.10 | 0.70 | 17.07 | 30 | 7.21 |  | 4.15 | 0.65 | 15.76 | 30 | 7.90 |  |
| I1 | 3.75 | 0.77 | 20.43 | 20 | 3.67 | √ |  |  |  |  |  |  |
| I2 | 3.75 | 0.77 | 20.43 | 20 | 3.67 | √ |  |  |  |  |  |  |
| I3 | 3.75 | 0.77 | 20.43 | 20 | 3.67 | √ |  |  |  |  |  |  |
| I4 | 3.75 | 0.77 | 20.43 | 20 | 3.67 | √ |  |  |  |  |  |  |
| I5 | 3.75 | 0.77 | 20.43 | 20 | 3.67 | √ |  |  |  |  |  |  |
| I6 | 3.75 | 0.77 | 20.43 | 20 | 3.67 | √ |  |  |  |  |  |  |
| I7 | 3.75 | 0.77 | 20.43 | 20 | 3.67 | √ |  |  |  |  |  |  |
| I8 | 3.75 | 0.77 | 20.43 | 20 | 3.67 | √ |  |  |  |  |  |  |
| I9 | 3.75 | 0.77 | 20.43 | 20 | 3.67 | √ |  |  |  |  |  |  |
| I10 | 3.75 | 0.77 | 20.43 | 20 | 3.67 | √ |  |  |  |  |  |  |
| I11 | 3.75 | 0.77 | 20.43 | 20 | 3.67 | √ |  |  |  |  |  |  |
| I12 | 3.75 | 0.77 | 20.43 | 20 | 3.67 | √ |  |  |  |  |  |  |
| I13 | 3.75 | 0.77 | 20.43 | 20 | 3.67 | √ |  |  |  |  |  |  |
| J1 | 4.50 | 0.50 | 11.11 | 50 | 20.25 |  | 4.50 | 0.50 | 11.11 | 50 | 20.25 |  |
| J2 | 4.15 | 0.73 | 17.50 | 35 | 8.30 |  | 4.15 | 0.73 | 17.50 | 35 | 8.30 |  |
| J3 | 3.50 | 0.81 | 23.03 | 10 | 1.52 | √ |  |  |  |  |  |  |
| J4 | 4.55 | 0.60 | 12.96 | 60 | 21.06 |  | 4.55 | 0.59 | 12.96 | 60 | 21.06 |  |
| J5 | 4.60 | 0.49 | 10.65 | 60 | 25.92 |  | 4.60 | 0.49 | 10.65 | 60 | 25.92 |  |
| J6 | 4.45 | 0.59 | 13.24 | 50 | 16.81 |  | 4.45 | 0.59 | 13.25 | 50 | 16.79 |  |
| J7 | 4.30 | 0.56 | 12.95 | 35 | 11.62 |  | 4.30 | 0.56 | 12.95 | 35 | 11.62 |  |
| K1 | 4.55 | 0.59 | 12.96 | 60 | 21.06 |  | 4.55 | 0.59 | 12.96 | 60 | 21.06 |  |
| K2 | 4.60 | 0.58 | 12.68 | 65 | 23.58 |  | 4.60 | 0.58 | 12.68 | 65 | 23.58 |  |
| K3 | 4.15 | 0.65 | 15.76 | 30 | 7.90 |  | 4.15 | 0.65 | 15.76 | 30 | 7.90 |  |
| K4 | 3.80 | 0.68 | 17.85 | 15 | 3.19 | √ |  |  |  |  |  |  |
| K5 | 4.10 | 0.62 | 15.23 | 25 | 6.73 |  | 4.10 | 0.62 | 15.23 | 25 | 6.73 |  |
| L1 | 4.65 | 0.73 | 9.90 | 30 | 14.00 | √ |  |  |  |  |  |  |
| L2 | 3.20 | 0.68 | 16.15 | 15 | 2.97 |  | 4.20 | 0.68 | 16.15 | 35 | 9.10 |  |
| L3 | 4.15 | 0.73 | 17.50 | 35 | 8.30 |  | 4.15 | 0.73 | 17.50 | 35 | 8.30 |  |
| L4 | 4.40 | 0.66 | 15.08 | 50 | 14.59 |  | 4.40 | 0.66 | 15.08 | 50 | 14.59 |  |
| L5 | 4.25 | 0.62 | 14.65 | 35 | 10.15 |  | 4.25 | 0.62 | 14.65 | 35 | 10.15 |  |
| M1 | 4.10 | 0.44 | 10.63 | 15 | 5.79 |  | 4.10 | 0.44 | 10.63 | 15 | 5.79 |  |
| M2 | 3.65 | 0.73 | 19.90 | 10 | 1.83 | √ |  |  |  |  |  |  |
| M3 | 3.65 | 0.73 | 19.90 | 10 | 1.83 | √ |  |  |  |  |  |  |
| M4 | 3.65 | 0.73 | 19.90 | 10 | 1.83 | √ |  |  |  |  |  |  |
| M5 | 3.65 | 0.73 | 19.90 | 10 | 1.83 | √ |  |  |  |  |  |  |
| M6 | 3.65 | 0.73 | 19.90 | 10 | 1.83 | √ |  |  |  |  |  |  |
| M7 | 3.65 | 0.73 | 19.90 | 10 | 1.83 | √ |  |  |  |  |  |  |
| M8 | 3.65 | 0.73 | 19.90 | 10 | 1.83 | √ |  |  |  |  |  |  |
| M9 | 3.65 | 0.73 | 19.90 | 10 | 1.83 | √ |  |  |  |  |  |  |
| M10 | 3.65 | 0.73 | 19.90 | 10 | 1.83 | √ |  |  |  |  |  |  |
| M11 | 3.65 | 0.73 | 19.90 | 10 | 1.83 | √ |  |  |  |  |  |  |
| N1 | 4.60 | 0.58 | 12.68 | 65 | 23.58 |  | 4.65 | 0.48 | 10.26 | 65 | 29.46 |  |
| N2 | 3.70 | 0.64 | 17.31 | 10 | 2.14 | √ |  |  |  |  |  |  |
| N3 | 4.45 | 0.67 | 15.03 | 55 | 16.28 |  | 4.50 | 0.59 | 13.14 | 55 | 18.84 |  |
| N4 | 3.70 | 0.64 | 17.31 | 10 | 2.14 | √ |  |  |  |  |  |  |
| N5 | 4.45 | 0.67 | 15.03 | 55 | 16.28 |  | 4.50 | 0.59 | 13.14 | 55 | 18.84 |  |
| N6 | 3.80 | 0.68 | 17.85 | 15 | 3.19 | √ |  |  |  |  |  |  |
| N7 | 3.80 | 0.68 | 17.84 | 15 | 3.20 | √ |  |  |  |  |  |  |
| N8 | 4.45 | 0.67 | 15.03 | 55 | 16.28 |  | 4.45 | 0.67 | 15.03 | 55 | 16.28 |  |
| N9 | 3.80 | 0.68 | 17.84 | 15 | 3.20 | √ |  |  |  |  |  |  |
| O1 | 3.75 | 0.62 | 16.60 | 10 | 2.26 | √ |  |  |  |  |  |  |
| O2 | 4.45 | 0.67 | 15.03 | 55 | 16.28 |  | 4.45 | 0.67 | 15.03 | 55 | 16.28 |  |
| O3 | 3.75 | 0.62 | 16.60 | 10 | 2.26 | √ |  |  |  |  |  |  |
| O4 | 4.55 | 0.59 | 12.96 | 60 | 21.06 |  | 4.45 | 0.59 | 12.96 | 60 | 20.60 |  |
| O5 | 4.60 | 0.58 | 12.68 | 65 | 23.58 |  | 4.60 | 0.58 | 12.68 | 65 | 23.58 |  |
| O6 | 3.70 | 0.64 | 17.31 | 10 | 2.14 | √ |  |  |  |  |  |  |
| O7 | 3.80 | 0.75 | 19.69 | 20 | 3.86 | √ |  |  |  |  |  |  |
| O8 | 3.65 | 0.57 | 15.68 | 5 | 1.16 | √ |  |  |  |  |  |  |
| O9 | 4.55 | 0.59 | 12.96 | 60 | 21.06 |  | 4.55 | 0.59 | 12.96 | 60 | 21.06 |  |
| O10 | 3.90 | 0.77 | 19.70 | 15 | 2.97 | √ |  |  |  |  |  |  |
| O11 | 4.70 | 0.71 | 19.30 | 15 | 3.65 | √ |  |  |  |  |  |  |
| O12 | 3.75 | 0.54 | 14.40 | 10 | 2.60 | √ |  |  |  |  |  |  |
| O13 | 3.95 | 0.74 | 18.73 | 15 | 3.16 | √ |  |  |  |  |  |  |
| O14 | 3.90 | 0.62 | 16.01 | 15 | 3.65 | √ |  |  |  |  |  |  |
| P1 | 3.85 | 0.65 | 16.98 | 15 | 3.40 | √ |  |  |  |  |  |  |
| P2 | 3.85 | 0.65 | 16.98 | 15 | 3.40 | √ |  |  |  |  |  |  |
| P3 | 3.85 | 0.65 | 16.98 | 15 | 3.40 | √ |  |  |  |  |  |  |
| P4 | 3.85 | 0.65 | 16.98 | 15 | 3.40 | √ |  |  |  |  |  |  |
| P5 | 3.85 | 0.65 | 16.98 | 15 | 3.40 | √ |  |  |  |  |  |  |
| P6 | 3.85 | 0.65 | 16.98 | 15 | 3.40 | √ |  |  |  |  |  |  |
| P7 | 3.85 | 0.65 | 16.98 | 15 | 3.40 | √ |  |  |  |  |  |  |
| P8 | 3.85 | 0.65 | 16.98 | 15 | 3.40 | √ |  |  |  |  |  |  |
| Q1 | 4.45 | 0.50 | 11.00 | 45 | 18.20 |  | 4.45 | 0.50 | 11.18 | 45 | 17.91 |  |
| Q2 | 4.60 | 0.49 | 10.65 | 60 | 25.92 |  | 4.60 | 0.49 | 10.65 | 60 | 25.92 |  |
| Q3 | 4.50 | 0.59 | 13.14 | 55 | 18.84 |  | 4.50 | 0.59 | 13.14 | 55 | 18.84 |  |
| Q4 | 4.50 | 0.59 | 13.14 | 55 | 18.84 |  | 4.50 | 0.59 | 13.14 | 55 | 18.84 |  |
| Q5 | 4.45 | 0.67 | 15.03 | 55 | 16.28 |  | 4.50 | 0.59 | 13.14 | 55 | 18.84 |  |
| Q6 | 3.80 | 0.81 | 21.38 | 15 | 2.67 | √ |  |  |  |  |  |  |
| Q7 | 2.45 | 0.59 | 23.25 | 45 | 4.74 |  | 4.50 | 0.50 | 11.11 | 50 | 20.25 |  |
| Q8 | 4.60 | 0.58 | 12.68 | 65 | 23.58 |  | 4.65 | 0.48 | 10.26 | 65 | 29.46 |  |
| Q9 | 4.55 | 0.59 | 12.96 | 60 | 21.06 |  | 4.60 | 0.49 | 10.65 | 60 | 25.92 |  |
| R1 | 4.35 | 0.57 | 13.16 | 40 | 13.22 |  | 4.40 | 0.49 | 11.13 | 40 | 15.81 |  |
| R2 | 4.50 | 0.50 | 11.11 | 50 | 20.25 |  | 4.50 | 0.50 | 11.11 | 50 | 20.25 |  |
| R3 | 4.55 | 0.59 | 12.96 | 60 | 21.06 |  | 4.60 | 0.49 | 10.65 | 60 | 25.92 |  |
| R4 | 4.40 | 0.49 | 11.13 | 40 | 15.81 |  | 4.40 | 0.49 | 11.13 | 40 | 15.81 |  |
